# Supplementary material for: Chromogenic Chemodosimeter Based on Capped Silica Particles to Detect Spermine and Spermidine
Source: Nanomaterials (Basel). 2021 Mar 23;11(3):818. doi: 10.3390/nano11030818 (PMC8004735; doi:10.3390/nano11030818)
Supplement: Supplementary file 1 [file nanomaterials-11-00818-s001.pdf]

Supplementary Material

Chromogenic Chemodosimeter Based on Capped Silica Particles to Detect Spermine and Spermidine

Mariana Barros [1], Alejandro López-Carrasco [1], Pedro Amorós [3*], Salvador Gil [1,2], Pablo Gaviña [1,2], Margarita Parra [1,2], Jamal El Haskouri [3], M. Carmen Terencio [1,4], Ana M. Costero [1,2*]

Contents

Table S1. Comparison of sensing properties of different Spm/Spd selective optical probes... 2

Figure S1. ^{1}H NMR compound **1** in CD_3CN. 4

Figure S2. Solid ^{13}C NMR spectra of MCM-41 after reacting with compound **1**. 4

Figure S3. DLS curves of (a) **MCM-41** and (b) **S1**. 5

Figure S4. TGA curve of the **S1** material. 5

Figure S5. ^{1}H NMR spectra of probe **1** (MCM-41 treated with compound **1**) and probe **1** in presence of Spd. The circled signals correspond to the liberated N-hydroxysuccinimide derivative........ 6

Figure S6. Fluorescence titration of **S1** with Spd 6

Figure S7. Calibration curve: emission at λ_{em}=550 nm (λ_{ex}= 525 nm) 7

Figure S8. Rhodamine 6G (λem = 550 nm, λex = 525 nm) delivery profiles from S1 particles in the absence and presence of different amines........ 7

Figure S9. Calibration curve of Rhodamine 6G in NaOH (0.5 M) and MeOH........ 8

Figure S10. Emission after disgregation in NaOH 0.5 M (275.6 at 550 nm) 8

Figure S11. Cell viability determined by the MTT assay after 2h-incubation of RAW 264,7 macrophages with different concentrations of S1, Spermine (Spm), Spermidine (Spd) and the mixture.

Table S1. Comparison of sensing properties of different Spm/Spd selective optical probes.

Probe	Detection technique	Selectivity	Limit of detection	Application	Reference
**MCM-41 nanoparticles functionalized with a N-hydroxysuccinimide derivative and loaded with rhodamine 6G**	Fluorescence turn on	For Spm and Spd	27 µM (Spm) 45 µM (Spd)	RAW 264.7 macrophages	This study
**Tyrosine functionalized gold nanoparticles**	Abs and fluorescence turn on	For Spm and Spd	136 and 636 pM (colorimetric) 6.2 and 5.3 nM (fluorimetric)	Human plasma and urine	Rawat *et al.*, 2017
**Gold nanoparticles based**	Abs	For Spm	10 ppb	Human urine	Jornet-Martinez *et al.*, 2014
**BODIPY functionalized gold nanoparticles**	Fluorescence turn on	For Spm and Spd	NA	Artificial urine	Kim *et al*, 2011
**Ag-Au/AgCl nanohybrid**	Fluorescence turn off	For Spm	0.87 nM	Human urine	Kuo *et al.*, 2018
**DNA aptamer coated gold nanoparticles**	Abs	For Spm	15.25 nM	Artificial and clinical urine	Tsoi *et al.*, 2017
**ssDNA-caped gold nanoparticles**	Abs	For Spm	13.9 nM	Human plasma and urine	De Liu *et al.*, 2013
**Pepsin gold nanoclusters (pepsin-Au NCs)**	Fluorescence turn off	For Spm	1.75 nM	Human plasma and urine	Bhamore *et al.*, 2019
**perylene diimide EA-PDI-Cu2+ complex**	Abs and fluorescence turn off	For Spm	86.3 nM (UV-vis) and 90 pM (fluorescence)	Urine and blood serum	Kumar *et al.*, 2019
**Tricyclic dihydropyrimidine-based multifunctional organicnanoparticles**	Fluorescence turn on	For Spd	3.2 nM	Human urine	Kaur *et al.*, 2017
**Au-decorated SiO2 NPs (AuSil), a metal (Au)-dielectric (SiO2) hybrid material**	Fluorescence turn on	For Spd	fM	NA	Bhaskar *et al.*, 2020

Rawat, K. A.; Bhamore, J. R.; Singhal, R. K.; Kailasa, S. K. Microwave assisted synthesis of tyrosine protected gold nanoparticles for dual (colorimetric and fluorimetric) detection of spermine and spermidine in biological samples. *Biosensors and Bioelectronics* **2017**, *88*, 71-77.

Jornet-Martinez, N.; González-Béjar, M.; Moliner-Martínez, Y.; Campins-Falco, P.; Pérez-Prieto, J. Sensitive and selective plasmonic assay for spermine as biomarker in human urine. *Analytical chemistry* **2014**, *86*, 1347-1351.

Kim, T. I.; Park, J.; Kim, Y. A Gold Nanoparticle-Based Fluorescence Turn-On Probe for Highly Sensitive Detection of Polyamines. *Chemistry–A European Journal* **2011**, *17*, 11978-11982.

Kuo, P. C.; Lien, C. W.; Mao, J. Y.; Unnikrishnan, B.; Chang, H. T.; Lin, H. J.; Huang, C. C. Detection of urinary spermine by using silver-gold/silver chloride nanozymes. *Analytica chimica acta* **2018**, *1009*, 89-97.

Tsoi, T. H.; Gu, Y. J.; Lo, W. S.; Wong, W. T.; Wong, W. T.; Ng, C. F.; Wong, K. L. Study of the Aggregation of DNA-Capped Gold Nanoparticles: A Smart and Flexible Aptasensor for Spermine Sensing. *ChemPlusChem* **2017**, *82*, 802-809.

De Liu, Z.; Zhu, H. Y.; Zhao, H. X.; Huang, C. Z. Highly selective colorimetric detection of spermine in biosamples on basis of the non-crosslinking aggregation of ssDNA-capped gold nanoparticles. *Talanta* **2013**, *106*, 255-260.

Bhamore, J. R.; Murthy, Z. V. P.; Kailasa, S. K. Fluorescence turn-off detection of spermine in biofluids using pepsin mediated synthesis of gold nanoclusters as a probe. *Journal of Molecular Liquids* **2019**, *280*, 18-24.

Kumar, K.; Kaur, S.; Kaur, S.; Bhargava, G.; Kumar, S.; & Singh, P. Perylene diimide–Cu 2+ based fluorescent nanoparticles for the detection of spermine in clinical and food samples: a step toward the development of a diagnostic kit as a POCT tool for spermine. *Journal of Materials Chemistry B* **2019**, *7*, 7218-7227.

Kaur, G.; Raj, T.; Singhal, S.; Kaur, N. Tricyclic dihydropyrimidine-based multifunctional organic nanoparticles for detection of Ag (I) ions and spermidine: real-time application. *Sensors and Actuators B: Chemical* **2018**, *255*, 424-432.

Bhaskar, S.; Kowshik, N. C. S.; Chandran, S. P.; Ramamurthy, S. S. Femtomolar Detection of Spermidine Using Au Decorated SiO2 Nanohybrid on Plasmon-Coupled Extended Cavity Nanointerface: A Smartphone-Based Fluorescence Dequenching Approach. *Langmuir* **2020**, *36*, 2865-2876.

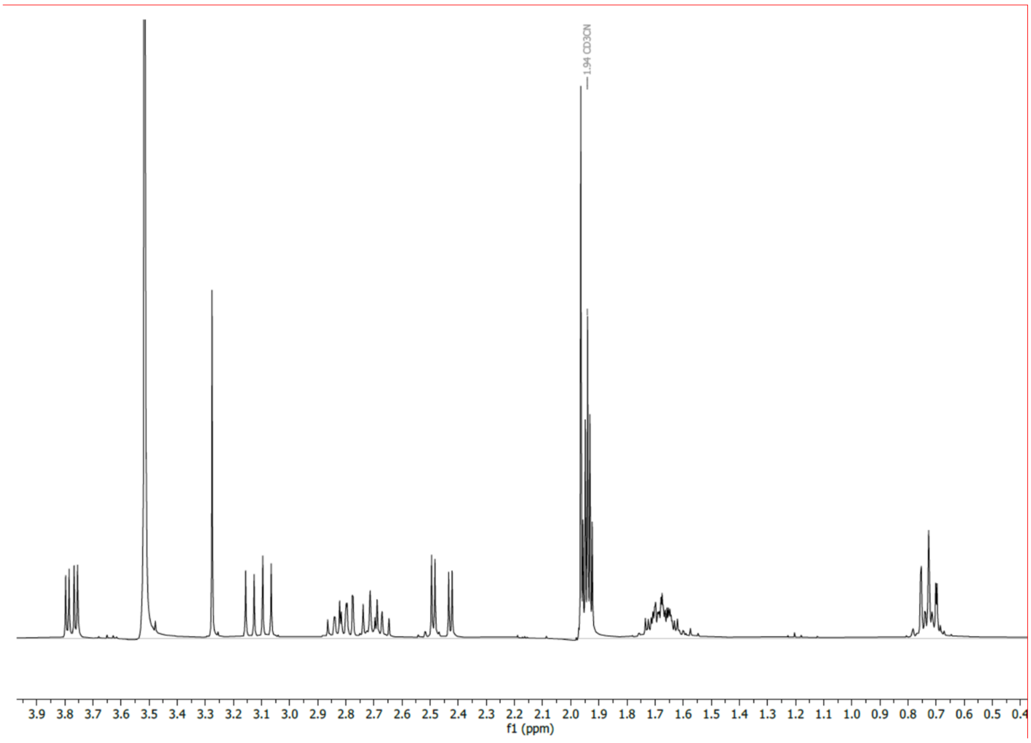

Figure S1. ^{1}H NMR compound **1** in CD_3CN.

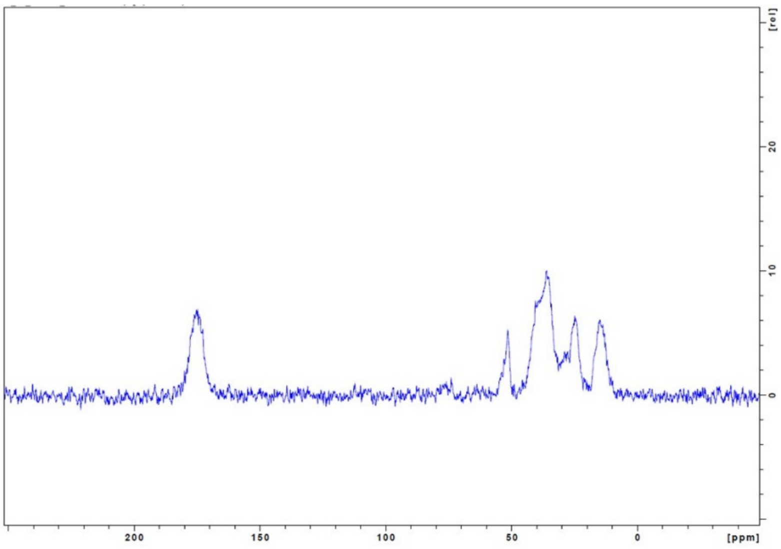

Figure S2. Solid ^{13}C NMR spectra of MCM-41 after reacting with compound **1**.

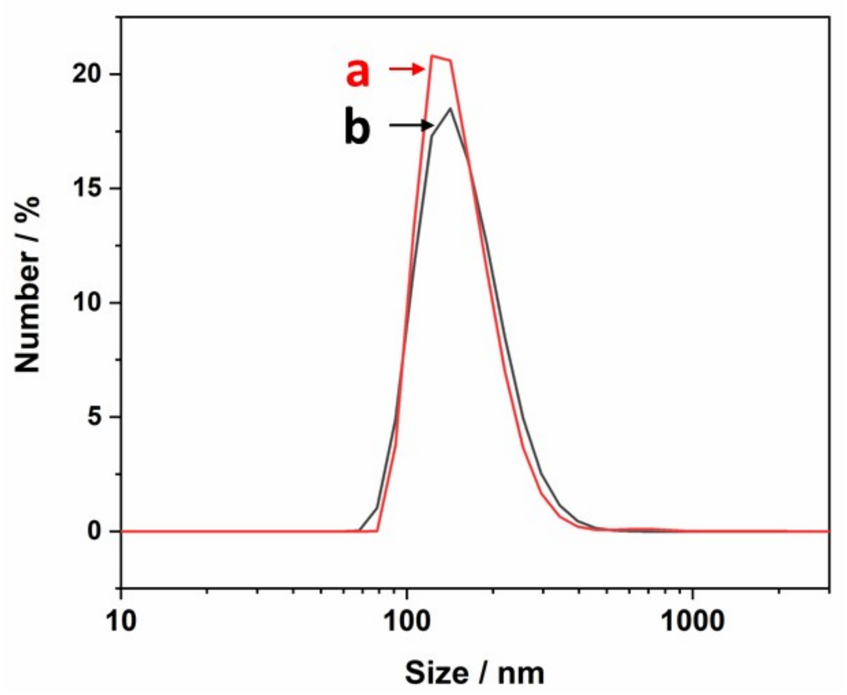

Figure S3. DLS curves of (a) MCM-41 and (b) S1.

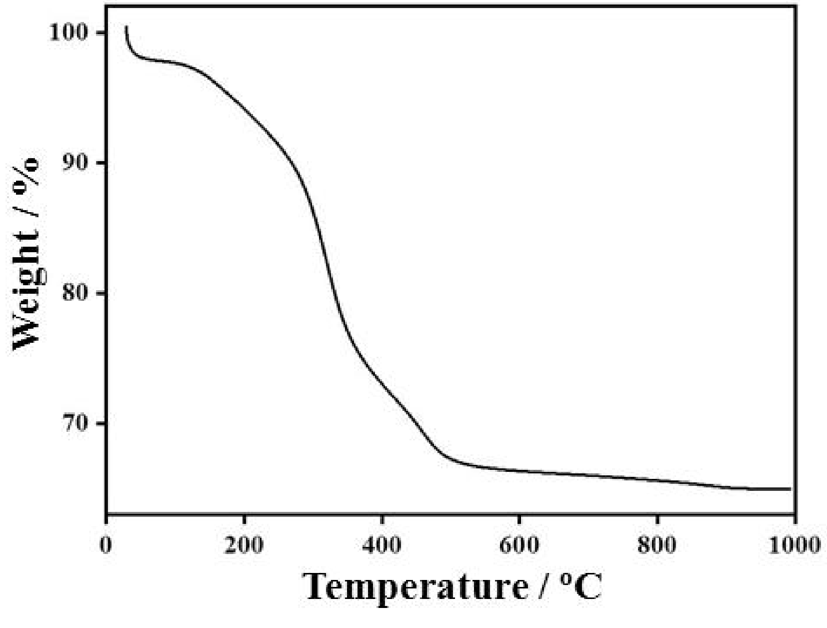

Figure S4. TGA curve of the S1 material.

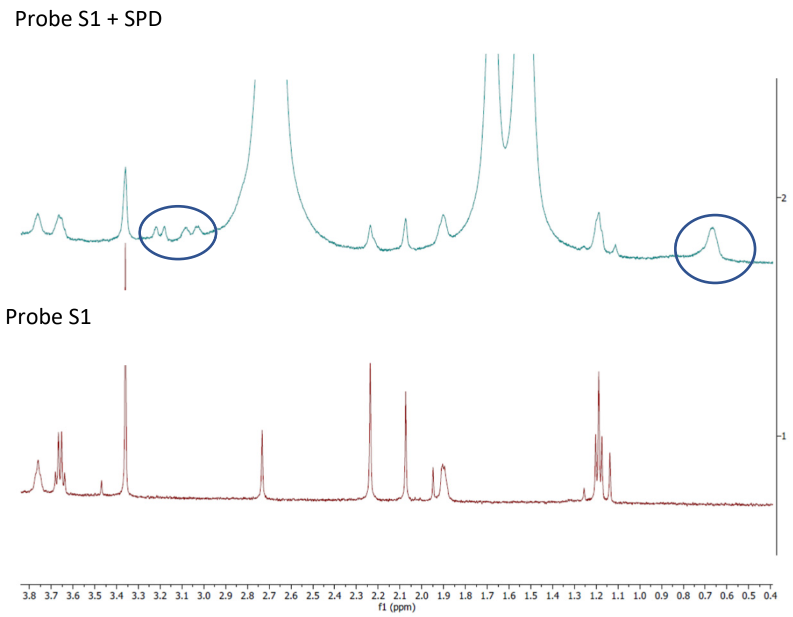

Figure S5. ^{1}H NMR spectra of probe **1** (MCM-41 treated with compound **1**) and probe **1** in presence of Spd. The circled signals correspond to the liberated N-hydroxysuccinimide derivative.

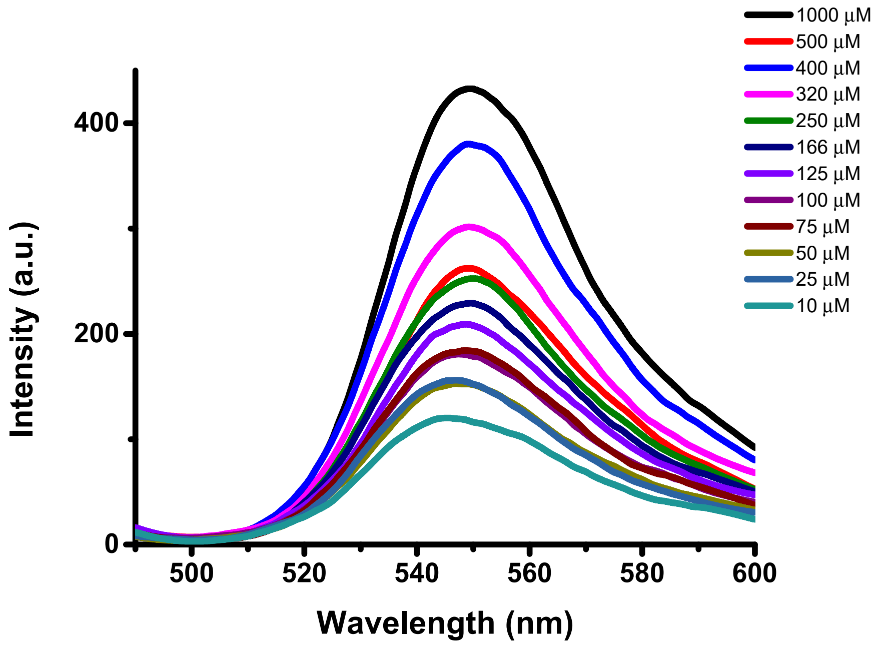

Figure S6. Fluorescence titration of **S1** with Spd

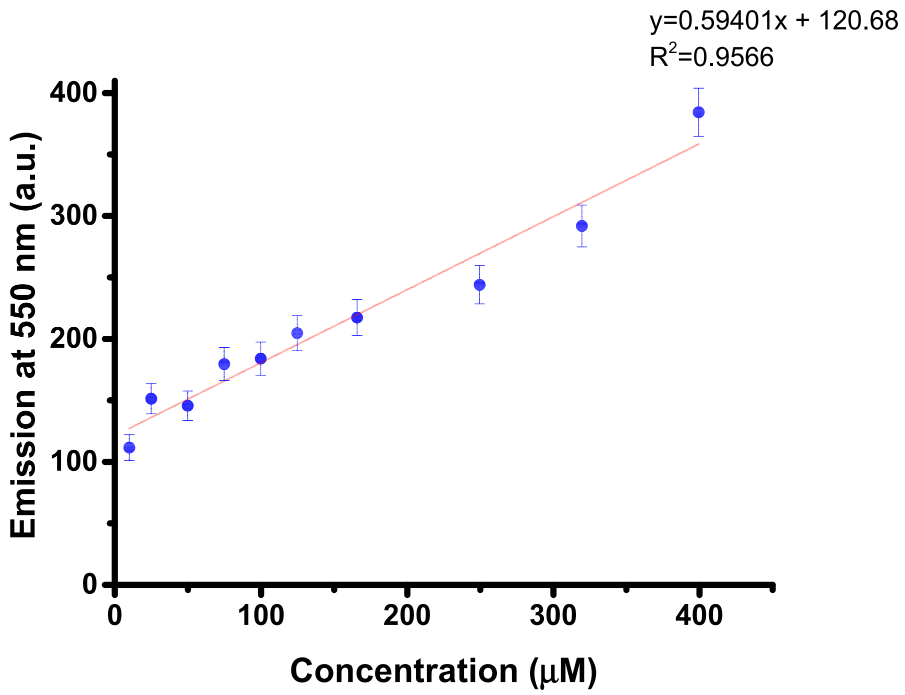

Figure S7. Calibration curve: emission at λ_{em}=550 nm (λ_{ex}= 525 nm)

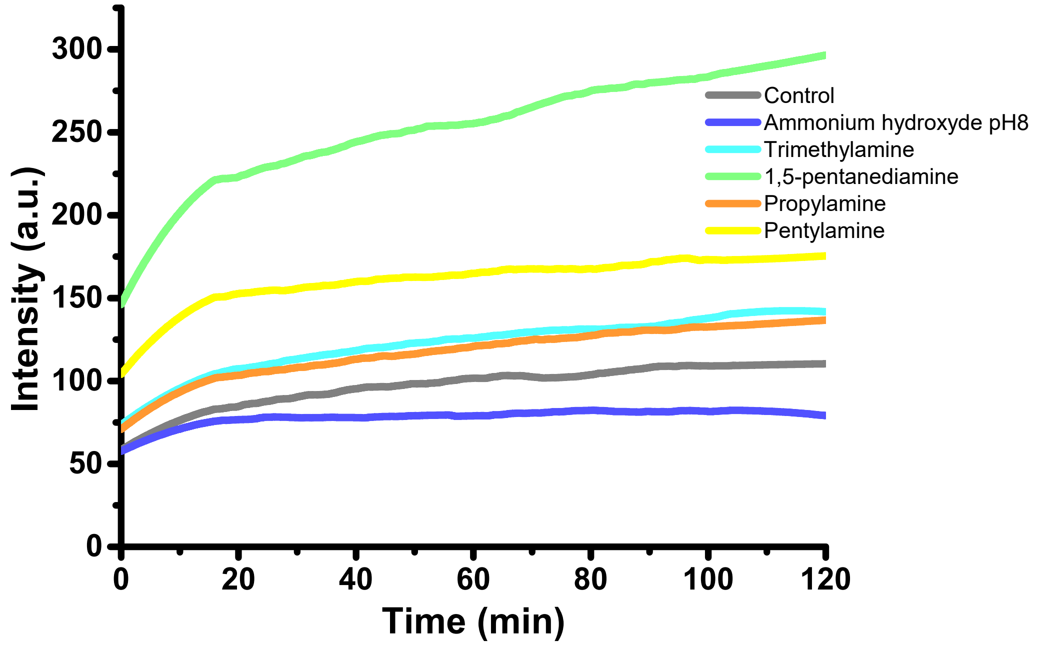

Figure S8. Rhodamine 6G (λem = 550 nm, λex = 525 nm) delivery profiles from S1 particles in the absence and presence of different amines.

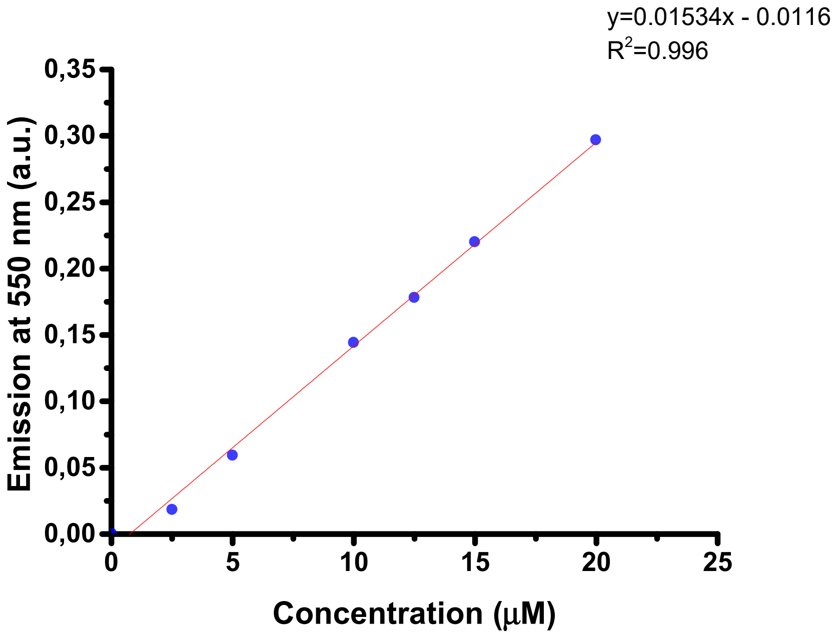

Figure S9. Calibration curve of Rhodamine 6G in NaOH (0.5 M) and MeOH

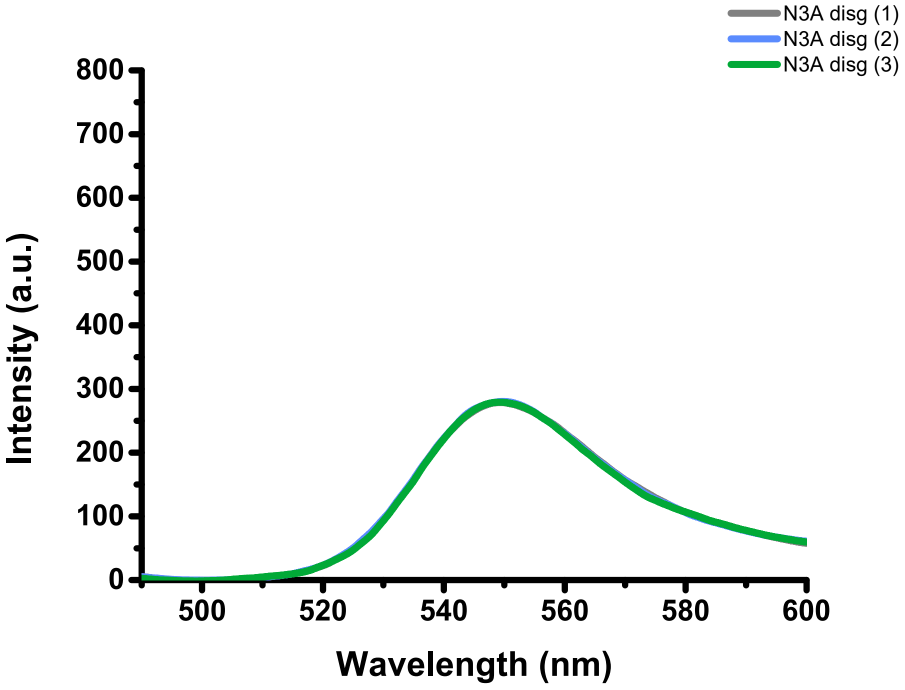

Figure S10. Emission after disgregation in NaOH 0.5 M (275.6 at 550 nm)

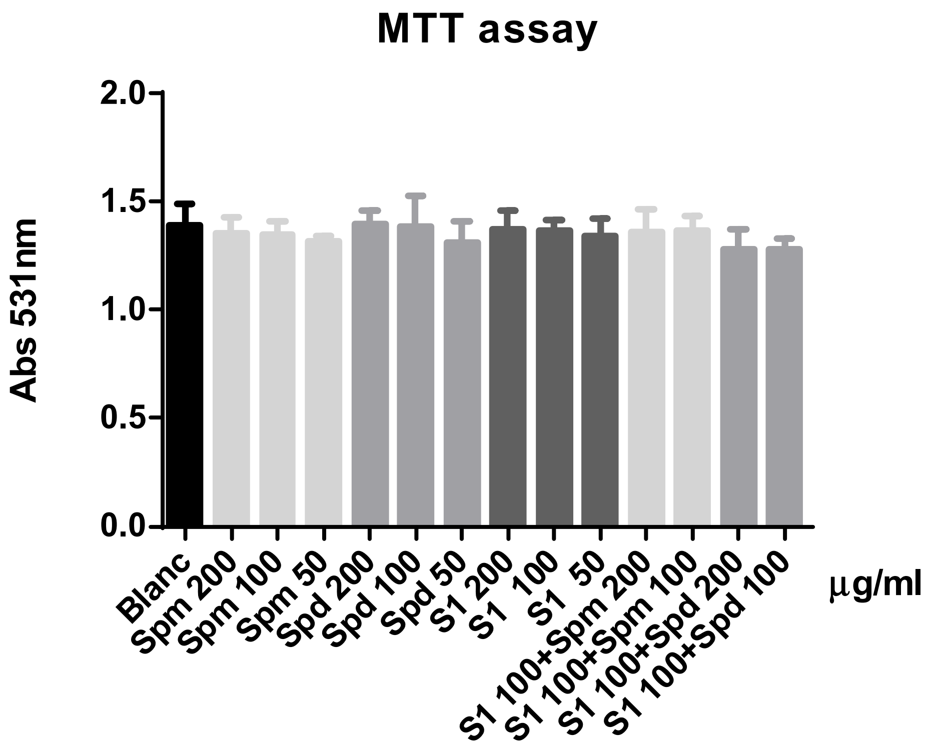

Figure S11. Cell viability determined by the MTT assay after 2h-incubation of RAW 264,7 macrophages with different concentrations of S1, Spermine (Spm), Spermidine (Spd) and the mixture.
